# Exosomes in pathogenesis, diagnosis, and therapy of ischemic stroke

**DOI:** 10.3389/fbioe.2022.980548

**Published:** 2022-12-16

**Authors:** Meiqi Jin, Shuxia Zhang, Mengchen Wang, Qiaoyu Li, Jiahui Ren, Yun Luo, Xiaobo Sun

**Affiliations:** ^1^ Institute of Medicinal Plant Development, Peking Union Medical College and Chinese Academy of Medical Science, Beijing, China; ^2^ Beijing Key Laboratory of Innovative Drug Discovery of Traditional Chinese Medicine (Natural Medicine) and Translational Medicine, Beijing, China; ^3^ Key Laboratory of Bioactive Substances and Resource Utilization of Chinese Herbal Medicine, Ministry of Education, Beijing, China; ^4^ NMPA Key Laboratory for Research and Evaluation of Pharmacovigilance, Beijing, China

**Keywords:** exosomes, ischemic stroke, blood vessels, neuron, inflammation, the blood-brain barrier (BBB), engineered exosomes

## Abstract

Ischemic stroke is one of the major contributors to death and disability worldwide. Thus, there is an urgent need to develop early brain tissue perfusion therapies following acute stroke and to enhance functional recovery in stroke survivors. The morbidity, therapy, and recovery processes are highly orchestrated interactions involving the brain with other tissues. Exosomes are natural and ideal mediators of intercellular information transfer and recognized as biomarkers for disease diagnosis and prognosis. Changes in exosome contents express throughout the physiological process. Accumulating evidence demonstrates the use of exosomes in exploring unknown cellular and molecular mechanisms of intercellular communication and organ homeostasis and indicates their potential role in ischemic stroke. Inspired by the unique properties of exosomes, this review focuses on the communication, diagnosis, and therapeutic role of various derived exosomes, and their development and challenges for the treatment of cerebral ischemic stroke.

## 1 Introduction

Exosomes were first discovered in sheep reticulocytes in 1983 (Zhang et al., 2020a). Johnstone et al. tracked transferrin receptors during the maturation of reticulocytes and found that the formation of exosomes is the mechanism for the loss of transferrin receptors in mature red blood cells (Johnstone et al., 1987). The International Society for Extracellular Vesicles uses the generic term extracellular vesicle (EV) to refer to particles that are naturally released from cells, enclosed in a lipid bilayer, and cannot replicate. Exosomes are considered subtypes of EVs. Some studies have compared EVs recovered using medium-speed centrifugation (referred to as large oncosomes, ectosomes, microvesicles, cell debris, or large or medium EVs) with those recovered using 100,000 × *g* ultracentrifugation (referred to as exosomes in the first four studies or small EVs in the last two); some of these studies used different density gradients for separation (Théry et al., 2018). Both prokaryotic and eukaryotic cells can release exosomes in either normal or pathological states. When secreted into the extracellular space, exosomes play a critical role in cell–cell communication by delivering bioactive substances between source and recipient cells. They also have targeting abilities and inherit specific characteristics. Thus, exosomes are of particular interest in biology because their formation involves a distinct extracellular regulatory process (Kalluri and LeBleu 2020). Under the conditions of specific physiological and pathological processes, some cell-derived exosomes undergo biological variations that include changes in the expression of proteins and nucleic acids. These are used to investigate mechanisms of biodegradation and biosynthesis, pathogenic injury, organ remodeling, and tissue repair (Sharma et al., 2019; Cheng et al., 2020). Although purification and identification of exosomes remain challenging, exciting discoveries concerning their molecular mechanisms in the field of stroke have emerged and their potential in diagnostic and therapeutic functions has been demonstrated (Yang et al., 2018a; Shin et al., 2020; Zhang et al., 2021a).

Extensive advances in the epidemiology, etiology, mechanism, and prognosis of stroke have been made; however, safe and effective treatments have not been developed for most patients. Disability and death are a huge burden on patients and their families worldwide (Zhang et al., 2020b). Cerebral stroke is an acute cerebrovascular disease that includes ischemic and hemorrhagic stroke. Ischemic stroke is the main type of cerebral stroke wherein blood cannot flow to the brain, resulting in the blockage of blood vessels, leading to brain tissue injury (Liang et al., 2016; Hong et al., 2019). When thrombogenesis blocks blood flow in the brain, the energy supply is disrupted, causing damage to the blood vessels and neuronal death. Blood–brain barrier (BBB) breakdown occurs in the first few hours after ischemic stroke and influences the permeability, induces secondary neuron inflammation, and accelerates the process of ischemic tissue damage (Liebner et al., 2018; Tuo et al., 2022). Meanwhile, an inflammatory reaction induced by gliocyte activation could also potentiate damage to the integrity of the BBB (Walsh et al., 2021). Primary cerebral ischemic treatment is to restore blood flow as soon as possible after the onset of symptoms. Alteplase is a recombinant tissue plasminogen activator (rt-PA) and only approved to treat cerebral ischemia stroke by the United States Food and Drug Administration. However, its therapeutic potential is limited by the hemolytic risk and short treatment window (4.5 h), with only a few patients benefitting from its use (Ishiguro et al., 2012; Bruch et al., 2019; Anfray et al., 2021). Therefore, finding a new therapeutic strategy against ischemic stroke is crucial.

Numerous studies have explained the mechanisms of ischemic stroke and ischemia/reperfusion (I/R) and offered several strategies for the use of exosomes for their diagnosis and treatment (Fei et al., 2021; Sun et al., 2021; Zhu et al., 2021). This review briefly describes exosomal biogenesis, collection methods, and communication. It investigates the role of exosomes in the diagnosis, neuroprotection, angiogenesis, anti-inflammation, and the BBB in ischemic stroke and focuses on the development of engineered exosomes. It reveals the mode of communication in various parts of the body in the ischemic stroke environment and the use of exosomes in repair or protective mechanisms. This review also reveals the challenges faced in these studies and provides new strategies for future research and therapeutic schemes for clinical treatment.

## 2 Exosomal composition and communication

### 2.1 Exosomal biogenesis and composition

**FIGURE 1:**
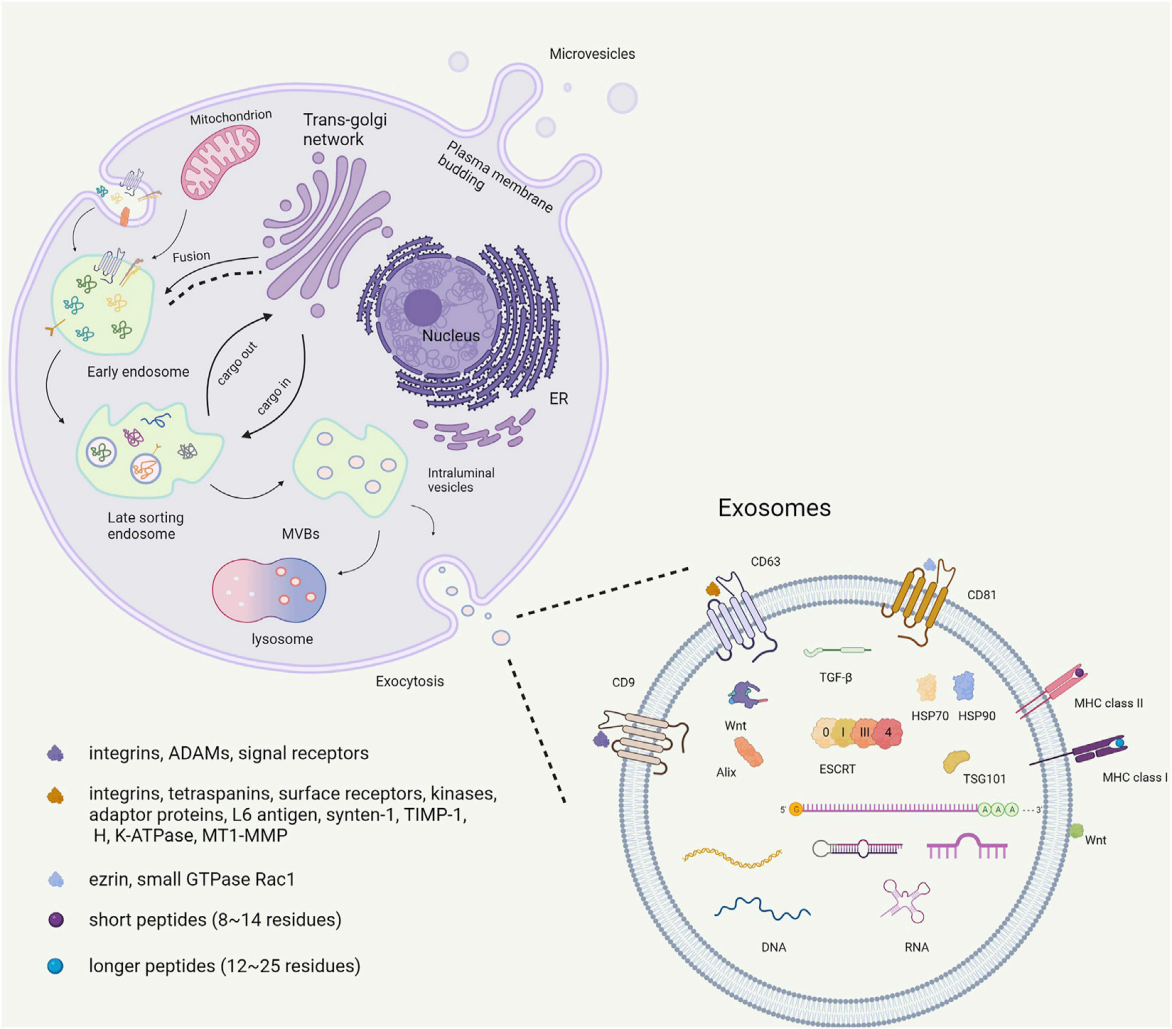
The biogenesis and contains of exosome.

Associating an EV with a particular biogenesis pathway remains extraordinarily difficult unless the EV is captured during release using live imaging techniques. Furthermore, using fluorescent exosome labeling and animal imaging technologies, the acting positions of exosomes can be dynamically tracked with the aim of providing technical support for increased accuracy in gene therapy. However, most studies suggest that exosomes are generated through a process involving the double invagination of the plasma membrane and formation of intracellular multivesicular bodies (MVBs) containing intraluminal vesicles (ILVs) (Théry et al., 2018). Exosomes are initially formed by endocytosis. The cell membrane is internalized to generate endosomes. Thereafter, many vesicles are formed within the endosome by internalizing a portion of the endosomal membrane. Finally, MVBs fuse with the cell membrane, releasing the intraluminal endoplasmic vesicles into the extracellular space to form exosomes (Gruenberg and van der Goot 2006). During this biogenesis process, exosomes carry multiple bioactive components, including proteins, nucleic acids, and lipids, and play a role in the biological functions especially in cellular communication. (Théry et al., 2002) (Figure 1). The heterogeneity of exosomes not only mirrors their size, content, and cellular origin but also reflects a regulated sorting mechanism. Ongoing technological and experimental advances are likely to yield valuable information regarding their heterogeneity and biological function(s), as well as enhance our ability to harness their therapeutic and diagnostic potential. Developing more standardized purification and analytical procedures to study exosomes will potentially lead to the uncovering of their functional heterogeneity (Kalluri and LeBleu 2020).

The exosome membrane is a phospholipid bilayer structure with a lipid composition, and it contains are similar to the plasmalemma component of the source cell. (Jeppesen et al., 2019). Exosomal proteins include membrane and intramembrane proteins. Membrane proteins are found in almost all exosomes, such as several tetraspanins (CD9, CD63, and CD81) and major histocompatibility complex class II (MHC II). Tetraspanins play important roles in normal (e.g., cell adhesion, motility, activation, and proliferation) and pathological conditions (e.g., metastasis and viral infection) (Yu et al., 2017). By contrast with many other cell surface proteins, CD9 does not have an obvious receptor function; it may participate in the organization of surface multiprotein complexes through association with other transmembrane molecules, including integrins, ADAMs, and signal receptors, thereby mediating various cellular and physiological functions (Liu et al., 2019a). CD63 interacts with many different proteins either directly or indirectly. Its interaction partners include integrins (α4β1, α3β1, α6β1, LFA-1, and β2), other tetraspanins (CD81, CD82, CD9, CD151), cell surface receptors (MHCII, CD3, FcεRI, and CXCR4), kinases (phosphatidylinositol 4-kinase and the Src family tyrosine kinases Lyn and Hck), adaptor proteins (AP-2, AP-3, and AP-4), and other proteins, including L6 antigen, syntenin-1, TIMP-1, H, K-ATPase, and MT1-MMP (Pols and Klumperman 2009). CD81 has an important function in the immune system. Proteins such as ezrin and the small GTPase Rac1 have been suggested to interact with the C-terminal domain of CD81, providing a potential link between tetraspanins and the cytoskeleton (Sala-Valdés et al., 2006). CD9, CD63, and CD81 are considered exosome markers. Exosomes are known to display two main types of MHC molecules: MHC I and II. MHC I is recognized by CD8^+^ T-cell and MHC II is recognized by CD4^+^ T-cell. MHC molecules can bind both self and non-self peptides. MHC I bind short (roughly 8–14 residues) peptides derived from intracellular proteins, such as phosphorylated peptides. MHC II molecules are primarily expressed in antigen-presenting cells, and bind longer peptides (roughly 12–25 residues) (Racle et al., 2019). Thus, interactions of MHC in exosomes with immune cells can provide novel immunotherapy strategies for ischemic stroke. Moreover, peripheral surface proteins include Wnt and are rich in extracellular matrix (ECM) proteins, which play a role in adhesion and signaling (Zhang et al., 2015; Lerner et al., 2020). Another class of extracellular factors outside the membrane includes transforming growth factor (TGF-β). In addition to external characterization, there is inclusion characterization, and exosomes contain unique proteins, including heat shock proteins (HSP70, HSP90), ESCRT complexes composed of ESCRT-0, ESCRT-1, ESCRT-2, ESCRT-3, and a class of exosomal scaffolding protein Alix (Pegtel and Gould 2019).

Exosomal nucleic acids contain DNA, mRNA, and non-coding RNA. An increasing number of researchers noted the function of mRNA and non-coding RNA in exosomes. The mRNA component carried by exosomes has been shown to enter the plasma membrane and can be translated into proteins (Valadi et al., 2007). Particularly, specific microRNAs (miRNAs), approximately 18–25 nucleotides long, are categorized as small non-coding RNA. They can control the expression of specific genes by binding to complementary sequences in the 3′ untranslated regions (UTRs) of target mRNA transcripts to inhibit or activate mRNA translation and transcription (Sun et al., 2018a; Isaac et al., 2021). miRNAs play a vital information transfer role in intercellular communication by transferring large amounts of biological cargo encapsulated in recipient cells to regulate posttranscriptional gene expression. miRNAs are also involved in the development of neurodegenerative diseases (Kojima et al., 2018), neuropsychiatric disorders (Xu et al., 2012), and tumors (Zhao et al., 2020).

### 2.2 Intercellular communication

Previously, exosomes were considered cellular debris. However, emerging research results in multiple fields have shown that it is a critical player in mediating intercellular communication (Lande et al., 2020). The biological process of exosomes include initiation, endocytosis, polycystic formation, and fusion with the plasma membrane; this process is precise at every step. mRNA, miRNAs, and other cargo in exosomes can be released by source cells to deliver messages to neighboring cells and distant cells, thereby regulating the function of recipient cells. Exosomes allow for the intercellular transport of proteins and RNA and are also capable of antigen presentation (Chen et al., 2019). They make contact with target cells in three ways: firstly, exosomal membrane proteins bind to recipient cell membrane proteins, activating intracellular signaling pathways in recipient cells. Secondly, exosomal membrane proteins are undetected on the source cell membrane. This indicates that, in an extracellular matrix, the sheared fragments produced by proteases cleave exosomal membrane proteins and could act as ligands to combine with receptors on the membrane and activate intracellular signaling pathways. Thirdly, the exosomal membrane and that of the recipient cell fuse directly, leading to the release of proteins, mRNA, and miRNA in their cargo (Zagrean et al., 2018). Thus, exosomes enable communication between neighboring cells and between source and distant cells through humoral circulation without direct contact (Koide et al., 2022). In several studies, the exosome-mediated transfer of miRNAs has explained the neurovascular unit (NVU) interaction in brain remodeling and cerebral ischemic protection.

## 3 Extraction and identification of exosomes

### 3.1 Method of exosome extraction

Exosomes that occur naturally or that are engineered require sensitive extraction and analysis techniques for subsequent research on ischemic stroke. Currently, the gold standard technique for isolation and purification of exosomes from biological fluids or cell supernatants is ultracentrifugation or a combination with ultrafiltration membranes (density gradient centrifugation) (Yan et al., 2019). Density gradient centrifugation has been performed on exosomes in a sample enriched with sucrose in a density range of 1.13–1.19 g/ml by ultracentrifugation (Wang et al., 2014). Immunomagnetic extraction is another innovative method of enrichment and extraction of exosomes. This method uses exosomal membranes to express specific proteins, which are separated and enriched based on the specific binding of antigens and antibodies and the magnetic properties of magnetic beads (Clayton et al., 2001). Therefore, the exosomes extracted using the immunomagnetic method are of high purity. This method is not limited by instruments and reagents and is suitable for use with urine, blood, cell medium, and other sample types (Cai et al., 2018). However, specificity is both an advantage and a disadvantage because exosomes secreted by different cells differ in the type of membrane protein expression as well. Specific antibody-modified immunomagnetic beads are not universally applicable when extracting exosomes from different cell sources. In experiments, several methods are often combined to save time and improve purity.

### 3.2 Physical characterization

Conventional optical microscopes with magnification limits close to the size of exosomes (a diameter of 30–100 nm) are important; however, they are not adequate for observation. The physical characterization of exosomes, including morphology, size, and distribution, is often measured by more precise microscopic techniques (Raposo and Stoorvogel 2013; Hong et al., 2019). Scanning electron microscopy (SEM), transmission electron microscopy (TEM), and atomic force microscopy (AFM) are reliable and widely used techniques. They are different in the manner in which they analyze exosomes. TEM allows for observation of the internal structure. SEM primarily allows observation of the morphology and has a stereoscopic sense; however, it is limited to the surface structure of exosomes (Pascucci et al., 2014; Tegegn et al., 2016). The advantage of AFM is that the samples are intact, and the requirements for sample analysis can be readily met compared with those of SEM and TEM, as exosomes can be directly analyzed in atmospheric and liquid environments. Dynamic light scattering can calculate the drug encapsulation rate of exosomes and test their stability in different environments before and after encapsulation. Furthermore, nanoparticle tracking analysis (NTA) allows simultaneous analysis of particle size and concentration of exosomes, and when the concentration is too low, NTA can satisfactorily perform the detection. Compared with other techniques, NTA guarantees the original state of the exosomes and has faster detection.

The most commonly used method for determining exosome purity is the quantification of the total protein amount and total particle number. and lipid amounts and the total number of particles. Thus, ratios of proteins: particles, lipids: particles or lipids: protein should be reported along with global quantification estimation estimates as a measure of purity and thus reliability of the quantity measure (Théry et al., 2018).

## 4 Pathogenesis and potential therapeutic role of exosomes in ischemic stroke

Ischemic stroke is caused by reduced cerebral blood flow and insufficient supply of oxygen to the brain tissue due to vascular embolism. The longer the condition persists, the more serious the brain damage. However, while treatments reopen the occluded cerebral vessels, the pathological damage to the ischemic tissues, blood vessels, and the nervous system is often further aggravated or even irreversible, and the clinical symptoms worsen, causing cerebral I/R injury. Exosomes are also part of this physiological process, allowing critical intercellular communication. Numerous researchers found that exosomes could cross the BBB and communicate using proteins, mRNA, or miRNA in the NVU to maintain the homeostasis of the central nervous system (CNS) (Forró et al., 2021). Furthermore, various derived exosomes repair injured tissues and exhibit anti-apoptotic, anti-inflammatory, and protection on nerves and vasculature. Table 1 summarizes the relevant studies on various derived exosomes in ischemic stroke.

**TABLE 1:** Summary of various exosomes in ischemic stroke.

Derived	Pre-treatment	Cargo	Change	Outcome	References
BMECs	OGD/R	-	-	Protect PC12 cells against OGD/R injury	Zeng et al. (2020)
BMECs	OGD	microRNA-134	Downregulated	Suppressed OLs apoptosis	Xiao et al. (2019)
MECs	MCAO mice	miR-542-3p	Downregulated	Prevented ischemia-induced glial cell inflammatory response	Cai et al. (2021)
	OGD	TLR4	Upregulated		
ASCs	OGD/RP	miR-22-3p	Upregulated	Alleviate brain ischemic injury	Zhang et al. (2021b)
Primary stem cell	OGD/R EA treated	miR-146b	Upregulated	Promotes the differentiation of endogenous neural stem cells improve neurological injury after ischemic stroke	Zhang et al. (2020b)
DPSCs	-	-	-	Alleviated neurological impairment	Li et al. (2020c)
iPSC-NPCs	-	-	-	Promoting the survival and growth of neurons	Li et al. (2021a)
USC	MCAO rats	miR-26a	-	Promoted both proliferation and neuronal differentiation of NSCs after OGD/R	Ling et al. (2020)
NSC	OGD	-	-	Neuroprotection against experimental stroke	Sun et al. (2019)
BV2	Treated with IL-4	miR-137	Upregulated	Attenuated neuronal apoptosis decreased infarct volume	Zhang et al. (2021c)
Astrocyte	OGD	-	-	Suppress autophagy	Pei et al. (2019)
				Ameliorate neuronal damage	
Astrocyte	OGD	miR-17-5p	Upregulated	Improved neurobehaviors	Du et al. (2021)
				Reduced neuronal apoptosis	
Astrocyte	OGD/R	miR-34c	Upregulated	Reduces neurological damage	Wu et al. (2020)
HUVECs	OGD	miR-1290	Upregulated	Protects neurons by attenuating apoptosis	Yue et al. (2019)
HUVECs	H/R	miR-21-3P	Downregulated	Inhibited neurons apoptosis	Jiang et al. (2018a)
Serum	Young rats	CD46	High expression	Attenuated synaptic dysfunction and improve post-stroke functional recovery	Zhang et al. (2021d)
ECEs	Ischemic rats	Proteins and miRNA	Altering miRNAs and target protein profiles	Promotes axonal growth of cortical neurons	Zhang et al. (2020c)
ADSCs	MCAO rat	miR-181-5p	Upregulated	Promote the angiogenesis of BMECs after OGD	Yang et al. (2018a)
BMSCs	-	miRNA-29-3p	-	Promoting angiogenesis	Hou et al. (2020)
				Suppressing neuronal apoptosis	
BMSCs	CXCR4 transfect	-	-	Promote the proliferation and tube formation for angiogenesis	Li et al. (2020d)
				Protecting brain endothelial	
MSCs	-	miR-126	Overexpressing	Enhance the survival and angiogenic function of H/R-injured EC	Pan et al. (2019)
MSCs	-	miR-132-3p	Overexpressing	Reducing cerebral vascular ROS production, BBB injury, and brain injury	Pan et al. (2020)
Microglial	OGD	miR-424-5p	Upregulated	Cell damage	Xie et al. (2020)
				Permeability of BMEC	
BV2	IL-4	miR-26a	Upregulated	Promoted the tube formation	Tian et al. (2019)
ADSCs	-	miR-30d-5p	Overexpressing	Suppression of autophagy	Jiang et al. (2018b)
				Reduced the OGD-innduce inflammatory response	
ADSCs	Hypoxic pre-treated	circ-Rps5	Upregulated	Improved cognitive function by reducing neuronal damage	Yang et al. (2022a)
SCs	-	-	-	Ameliorate brain injury cause by cerebral I/R	Zhang et al. (2021e)
BMSCs	Hypoxia preconditioning	-	-	Alleviating OGD/R-induced injury	Yu et al. (2021)
				Promoting the anti-inflammatory polarization of microglia	
BMECs	-	-	-	I/R injury-induced M1-polarized microglia could be shifted toward M2 phenotype	Liu et al. (2021)
BMSCs	Hypoxic pre-treated	-	-	Neuroprotective effects against NLRP3 inflammasom-mediated pyroptosis	Kang et al. (2021)
hUCMSCs		miR-26b-5p	-	Repress M1 polarization of microglia by targeting CH25H to inactivate the TLR pathway	Li et al. (2020a)
hWJ-MSC	-	-	-	Reduced microglia-mediated neuroinflammation	Thomi et al. (2019)
Plasma	melatonin-treated	-	The miRNA profiles changed	Decreased the infarct volume	Wang et al. (2020)
				Reduces the secretion of inflammatory cytokines	
				Alleviate inflammation	
Microglial	GW4869 treated	-	-	Reversed ischemia-induced microglial activation, inflammatory response	Gao et al. (2020)
Cortical neurons	OGD	miR-181c-3p	Downregulated	Decreased astrocyte expression of CXCL1 and inflammatory factors	Song et al., 2019a
BV2	Treated with IL-4	miR-124	Knockdown	Attenuated ischemic brain	Song et al., 2019b
				Promoted neuronal survival	
bEnd.3	OGD	Protein and miRNA	Changes of exosomal miRNA and surface protein profiles	Provide new therapeutic targets for BBB protection in ischemic stroke	Yang et al. (2021b)
Macrophage	-	-	-	Cross the BBB	Yuan et al. (2017)
				Deliver BDNF to the brain	
Neuron	-	miR-132	-	Maintain brain vascular integrity	Xu et al. (2017)
ECFCs	-	-	-	Increased TJ protein expression	Gao et al. (2018)
				Contribute to BBB integrity	
MSCs	-	-	-	Improve BBB integrity	Williams et al. (2020)
NSCs	-	-	-	Protect the integrity of the blood–brain barrier	Webb et al. (2018)

### 4.1 Neuroprotection and nerve regeneration

A stroke is among the leading causes of death, and post-stroke neurological disorders are the leading cause of disability worldwide (Liang et al., 2017). Following the onset of ischemic stroke, nerve injury worsens with prolonged ischemia. Moreover, the effect of cerebral I/R injury on neurological damage and brain dysfunction is severe. Stem cells have multiple differentiation potentials and differentiation and developmental plasticity. In previous studies, conditioned medium derived from various stem cells was shown to be effective in treating I/R injury (Mathew et al., 2019; Li et al., 2020a; Lee et al., 2021; Tian et al., 2021). Studies have indicated that bone marrow mesenchymal stem cell (BMSC)-derived exosomes could increase neuron viability in oxygen-glucose deprivation/reperfusion (OGD/R) by reducing NLRP3 inflammatory vesicle-mediated scorch death *via* the promotion of AMPK-dependent autophagic flux (Zeng et al., 2020). In a study, the level of miRNA-134 from BMSC-derived exosomes decreased while brain microvascular endothelial cells (BMECs) were disposed of with OGD. Thereafter, the OGD-disposed oligodendroglia cells (OLs) were treated with BMECs. The results showed that miR-134 inhibitors exacerbated the changes in the expression of the procaspase-8- and caspase-8-cleaved product proteins, which was caused by ODG (Xiao et al., 2019). Mesenchymal stem cells (MSCs) can directly affect the function of brain parenchymal cells *via* MSC-exos (Cai et al., 2021); and Zhang et al. showed that when adipose-derived MSC (ASC)-exos and neurons were co-cultured, miR-22–3p in ASC-exos increased, thus increasing neuron viability *in vitro* and alleviating nerve injury (Zhang et al., 2021a). In addition, tail vein injections of several types of stem cell-free derived exosomes for treatment of middle cerebral artery occlusion (MCAO)/R in mice could promote neural function recovery (Sun et al., 2019; Zhang et al., 2020c; Ling et al., 2020; Li et al., 2021a; Li et al., 2021b; Zhang et al., 2021b). Moreover, the level of astrocyte-derived exosomes, including miR-3c and miR-17–5p, could be used to target TLR7 and BINP2, further decreasing neuronal damage, reducing apoptosis and oxidation, increasing neuronal activity, and improving neurobehaviors (Pei et al., 2019; Wu et al., 2020; Du et al., 2021). Endothelial cell-derived exosomes, such as those from human umbilical vein endothelial cells (HUVECs) and brain-derived endothelial cells (bEnd.3), could influence I/R injury. The miRNA expression of hypoxia/reoxygenation (H/R)-treated HUVECs changes considerably, in that 249 and 104 miRNAs were downregulated and upregulated, respectively. Further studies suggested that miR-12–3p *via* HUVEC-exos could protect neurons against H/R apoptosis (Jiang et al., 2018a). Through the proteomics analysis of exosomes, a study found that pro-inflammatory mediators (C1q, C3a, and C3b) in serum exosomes increase whereas the exosomal levels of CD46, a C3b/C4b-inactivating factor, decrease with age. The microglial expression of C3a, C3b, and the C3a receptor (C3aR) increased after treatment with aged rat-derived exosomes. By replacing aging exosomes with young exosomes, it was possible to reverse the decline of synaptic and neurological functions and deliver therapeutic benefits after stroke (Zhang et al., 2021c). Non-ischemic and ischemic cerebral endothelial cell-derived exosomes facilitate axonal growth by altering miRNAs and their target protein profiles in recipient neurons (Zhang et al., 2020a).

### 4.2 Vascular protection and angiogenesis

It was initially thought that stem cells could accumulate in damaged tissues and replace damaged cells by self-renewal and directed differentiation. Recent studies showed that the tissue repair and regenerative functions performed by stem cells are mediated by their paracrine effects at stem cells that are less differentiated and unstable at the site of injury (Rong et al., 2019). There is evidence that the paracrine role of stem cells may be an important mechanism for their function in angiogenesis. Cerebral I/R could change the metabolism and function of endothelial cells (ECs), which could trigger EC damage, which may lead to cell death, and multiple signaling pathways. Therefore, the repair of damaged ECs and the promotion of angiogenesis in the damaged area are of vital importance in cerebral ischemia-induced vascular injury. Adipose-derived stem cells (ADSCs) promote cerebral blood vessel remodeling. Similarly, ADSC-derived exosomes could act on the miRNA-181b/TRPM7 axis to improve injury of ECs subjected to OGD/R and ameliorate mobility and angiogenesis of BMECs; therefore, miR-181–5p contributes to angiogenesis (Yang et al., 2018b). MSCs are different from other stem cells in that they are primarily found in connective tissue and interstitial organs. In addition, structural and functional alterations in the brain microvasculature might be major barriers to adequate reperfusion of cerebral ischemia (Cipolla 2021; Yang et al., 2022a). Hou et al. found that miRNA-29–3p in BMSC-exos was downregulated after BMSCs were subjected to OGD. Additionally, PTEN was upregulated and angiogenesis decreased in MCAO rats (Hou et al., 2020). Li et al. transfected BMSCs with lentivirus encoded by CXCR4. Thereafter, BMSC-exos^CXCR4^ was injected into the ipsilateral lateral ventricle of MCAO model rat brain. The results indicated that BMSC-exo ^CXCR4^ could attenuate the activation of the Wnt-3a/β-catenin pathway, achieve anti-apoptosis, and promote the proliferation and tube formation of microvascular ECs (Li et al., 2020a). Moreover, miR-126 is an important regulator of EC functions and angiogenesis. MSC-exo upregulate the level of VEGF, EGF, PDGF, and bFGF in H/R-injured ECs *via* delivery of miR-126, activate PI3K/Akt/eNOS pathway, and promote angiogenesis (Pan et al., 2019). Similarly, MSC-exo ^miR−132−3p^ has been shown to act on the PI3K signaling pathway, have anti-apoptotic effects, and improve the function of oxidative stress-affected ECs (Pan et al., 2020). Except for the H/R-injured condition, polarized BV2 microglial cells also show pro-angiogenesis effects (Xie et al., 2020). Tian et al. demonstrated the polarization of microglia using LPS and interleukin 4 (IL-4). The transfer of polarized BV2 cells was performed by intravenous injection into MCAO mice. Thereafter, the expression of BV2-exo miRNA under different conditions was compared using miRNA microarray technology. The study found that miRNA-26a contains more IL-4-polarized BV2 cell-derived exosomes. Moreover, IL-4-polarized BV2 cells promoted tube formation of ECs by secreting exosomes and had a therapeutic effect on stroke (Tian et al., 2019).

### 4.3 Inflammation reaction

I/R of the CNS could elicit an inflammatory response that stimulates the innate immune system to activate a series of inflammatory cascades (Sun et al., 2020). The immune cells that initially respond are microglia. They are normally quiescent, and when they are activated by damage, they become immune effector cells of the CNS (Choi et al., 2009; Dixon et al., 2021). In fact, microglia are activated to M1 phenotypes, typically releasing pro-inflammatory mediators and exacerbating brain injury. Switching the polarization of microglia from the pro-inflammatory M1 phenotype to the anti-inflammatory M2 phenotype is a promising therapeutic strategy for ischemic stroke (Yang et al., 2011; Zheng et al., 2019). Neutrophils exacerbate oxidative stress and BBB damage and participate in the pathological responses to injury and inflammation (Kolaczkowska and Kubes 2013). Hence, mitigating the immune-mediated inflammatory response is a crucial goal for ischemic stroke treatment. In acute ischemic stroke, the expression of inflammation factors increases and that of miR-30days-5p decreases in animal and patient models. Jiang et al. demonstrated that miR-30days-5p enhances ADSC-exos, inhibiting autophagy-mediated microglia polarization, thereby preventing cerebral injury (Jiang et al., 2018b). In contrast to previous studies, circular RNAs (circRNAs) in exosomes also mediate biological mechanisms *via* gene regulation. miR-124–3p and SIRT7 are circ-Rps5 downstream targets, while hypoxia pretreatment with ADSC-exo^circ-Rps5^ could shift microglia from an M1 to M2 phenotype in the hippocampus, decreasing MCAO-induced inflammatory factor (IL-6, IL-1β, and TNF-α) expression (Yang et al., 2022b). Likewise, stem cells (Zhang et al., 2021c), BMSCs (Kang et al., 2021; Liu et al., 2021; Yu et al., 2021), human umbilical cord MSC-derived exosomes (Li et al., 2020b), human Wharton’s jelly MSCs (Thomi et al., 2019), and the plasma-derived exosomes (Wang et al., 2020) can ameliorate cerebral I/R injury attributed to the modulation of microglia polarization. Additionally, glutaminase 1 (GLS1), a mitochondrial enzyme, causes chronic neuroinflammation, learning deficits, and synaptic dysfunctions in transgenic animal models. Gao et al. observed that GLS1-mediated exosome release might play a key role in the formation of a neuroinflammatory microenvironment (Gao et al., 2020). Besides microglia, inflammation factors released by astrocytes also have a profound impact on the inflammatory response in cerebral ischemia. Song et al. investigated whether the *CXCL1* gene was upregulated in ischemia brain injury and if it promoted an inflammatory response in MCAO rats. Subsequent studies found that exosomes derived from cortical neurons that underwent OGD decrease the expression of *CXCL1* and inflammatory factors in astrocytes, following delivery of miR-181c-3p *via* exosomes (Song et al., 2019a). Alternatively, M2 microglia-derived exosomes attenuate ischemic brain injury and promote neuronal survival *via* exosomal miR-124 and its downstream target, USP14 (Song et al., 2019b). Proximity barcoding assay experiments showed that the numbers of bEnd.3-derived exosomes carrying various proteins (bFGF, CD146, EPHA2, ABCB5, and ITGB2) increase markedly during ischemia. Such proteins are related to angiogenesis, cell proliferation, and cell inflammation (Yang et al., 2021a).

### 4.4 Crossing and maintaining the BBB

As part of the NVU, the BBB is a dynamic regulatory boundary that controls and limits the exchange of molecules, ions, and cells between blood and the CNS (Yang et al., 2019). The BBB has a massive impact on maintaining the homeostatic microenvironment of the CNS and normal neuronal function. Following an ischemic stroke, the structural integrity of the BBB is affected, leading to a substantial increase in the paracellular permeability in the cerebral microvasculature, a noteworthy pathological characteristic of ischemic stroke (Jiang Y. et al., 2018). Furthermore, an impaired BBB aggravates cerebral injury progression and increases hemorrhage risk, which leads to poor patient outcomes and limits the use of tPA for treatment (Liu and Chopp 2016). Yuan et al. first demonstrated that exosomes do not necessarily have to be modified to penetrate the BBB in mammals (Yuan et al., 2017). Notably, native macrophage-derived exosomes interact with BMECs and regulate intercellular adhesion molecule-1 (ICAM-1), whose expression plays a key role in BBB support (Bendorius et al., 2018).

Neurons can regulate brain vascular integrity. A study conducted by Xu et al. showed that miR-132 functions as an intercellular signal, mediating neural regulation of brain vascular integrity, and indicated that neuronal exosomes are a novel communication mechanism for the brain (Xu et al., 2017). Moreover, a marker of BBB disruption is disruption of the tight junction (TJ) protein complex. Hypoxia pretreatment with endothelial colony-forming cell (ECFC)-derived exosomes increase TJ protein expression and target the PTEN/AKT signaling pathway; thus, the study showed that exosomes derived from ECFCs contribute to BBB integrity (Gao et al., 2018). Naturally, MSC- and neural stem cell derived exosomes may be neuroprotective, decreasing the severity of brain injury in addition to maintaining the BBB integrity (Webb et al., 2018; Williams et al., 2020). Vascular protection and revascularization in ischemic stroke diseases are different from other disease conditions, and the treatment of cerebral ischemia is usually concerned with increasing collateral circulation and maintaining CNS homeostasis rather than the peripheral (Monteforte et al., 2017). Simultaneously, several studies have demonstrated the unique advantage of vascular endothelium-derived exosomes in a variety of diseases and in crossing the BBB up to the CNS, thus focusing on cerebral ischemia.

## 5 Diagnosis of ischemic stroke

Based on the European Stroke Organization’s guidelines on intravenous thrombolysis for acute ischemic stroke, the use of rt-PA is conditional at onset depending on medical history and other parameters. Moreover, the type of the drug, tenecteplase-tPA or rt-PA, and the dose to be administered is determined according to differing degrees of onset. The main diagnostic tool for cerebral stroke is advanced imaging, which is utilized to determine whether the conditions for mechanical embolization are satisfied. If an acute stroke is to be treated with intravenous thrombolysis based on the current guidelines, a rapid, sensitive, and accurate diagnostic tool is required for such a short therapeutic window. Meanwhile, the profile of exosomes in blood, urine, and other media have considerable differences amongst patients with a particular disease and healthy individuals and can be easily analyzed for assessing disease risk (Yang et al., 2022c). Based on the above pathogenesis research, not all miRNAs with differential expression can be used as diagnostic biomarkers; however, those that are should be specific and stable.

Exosomes are secreted by all types of cells and are present in biological fluids, making their sampling appealing for tracking disease progression in liquid biopsies. Therefore, exosomes have diagnostic abilities as potential biomarkers, making distinct stages of ischemic stroke easier to diagnose based on differences in the levels of miRNA. The level of miR-134 in plasma exosomes in patients with acute ischemic stroke is higher than in normal controls, and infarct volume is positively associated with a worse prognosis in patients with stroke (Zhou et al., 2018). miRNA expression changes throughout the various phases of the stroke, including during the time of early symptoms, symptom appearance, and prognosis. During pathogenesis [comprising the hyperacute phase IS (HIS, <6 h), acute ischemic stroke (one to three and 4–7 days), subacute phase IS (SIS, 8–14 days), and recovery phase (RIS, >14 days)] of cerebral ischemia, the expression of miRNA in plasma varies in clinical investigations, i.e., the expression of mi-R-30a-5p, miR-422a, miR-21–5p, and miR-1256-2-3p were found to be different in the four phases (Chen et al., 2015; Kerr et al., 2018; Wang et al., 2018). Similarly, compared with the control group, the expression of miR-223 in serum exosomes of patients with acute ischemic stroke was notably upregulated. Moreover, exosomal miR-223 expression is higher in stroke patients with poor outcomes than in those with favorable outcomes (Chen et al., 2017), showing positive correlation with NIH Stroke Scale/Score. Moreover, transient ischemic attack and permanent cerebral ischemia are different. Three hours after permanent cerebral ischemia, a rapid reduction in the level of serum exosomal miR-126 occurs, and it returns to normal after 24 h (Chen et al., 2015). In addition, patients with large artery atherosclerosis show the lowest serum exosome miR-152–3p levels compared with those with small vessel occlusion, cardiac embolism, and stroke of undetermined etiology. Moreover, the level of miR-152–3p in serum exosomes is lower in acute ischemic stroke than in the chronic phase (Song et al., 2020).

Furthermore, atherosclerosis can promote thrombosis and is strongly associated with acute cerebrovascular morbidity; its progression involves exosomes delivering bioactive messages. miR-21, miR-29, miR-126, miR-133, miR-146, and miR-155 in EC-derived exosomes may act as functional biomarkers to diagnose and predict the outcomes of atherosclerosis (Hulsmans and Holvoet 2013; Cervio et al., 2015; Lu et al., 2019). Moreover, growth arrest and DNA damage-inducible protein-34 (GADD34) has opposing effects on different stimulus-induced cell apoptotic events in many diseases affecting the nervous system. There is an increase in GADD34 levels in plasma exosomes of cerebral ischemic rats, indicating that exosome GADD34 could be used as a diagnostic biomarker and therapeutic target in ischemic strokes (Yang et al., 2021b). Therefore, based on the combination of these specific miRNAs and proteins within the group, it could be recognized as potential biomarkers of exosomes to diagnose ischemic stroke.

## 6 Development of engineered exosomes

Natural exosomes express transmembrane proteins and membrane-anchored proteins that allow them to be biocompatible. Exosomes as natural vehicles can achieve the objectives of nucleic acid delivery and drug targeting across physiological barriers and have advantages of lower immunogenicity and toxicity and favorable pharmacokinetics (Song Y. et al., 2019; Li Y. J. et al., 2021). However, the short half-life (t_1/2_) and weak targeting ability of exosomes limit their clinical application; engineered exosomes are capable of breaking through these limitations (Han et al., 2019). By engineering exosomes, we can impart additional functionality to the exosomes with the aim of enabling *in vivo* imaging and tracking, which facilitates the understanding of their fate *in vivo*, including the uptake mechanisms and biodistribution. Exosome engineering can significantly promote the application of exosomes for therapy and targeted drug delivery in various brain pathologies. We plan to focus on the availability of engineered exosomes as delivery nanotechnologies and in *in vivo* imaging and tracking next. Table 2 summarizes the relevant studies on engineered exosomes in the brain.

**TABLE 2:** Summary of engineered exosomes in ischemic stroke.

Derived	Engineered method	Outcome	References
Whole blood of SD rats	Que loaded and mAb GAP43 conjugated	Targeting and therapeutic drug delivery system	Guo et al. (2021)
Macrophage	Curcumin loaded	Accumulated Ex-cur in ischemic regions to reduce ROS accumulation	He et al. (2020)
MESC	Curcumin loaded	Neurovascular restoration following I/R injury	Kalani et al. (2016)
Macrophage	Edaravone Loaded	Improved the biovailability of Edv and prolonged	Li et al. (2020b)
Mesenchymal cells	Transferrin combined and enkephalin packaged	Promote neurological recovery after stroke	Liu et al. (2019b)
ADSC	PEDF overexpressed	Activating autophagy and suppressing neuronal apoptosis	Huang et al. (2018)
BM-MSC	Modified exosomes with RVG fused to exosomal protein Lamp2b	Utilized therapeutically for the targeted delivery of gene drugs to the brain	Yang et al. (2017)
HEK293	RVG peptide on the surface and loaded NGF	Neuron targeting and NGF was delivered in to ischemic cortex	Yang et al. (2020)
MSC	c (RGDyK) peptide was conjugated and cholesterol-modified miR-210 loaded	Targets the lesion region of the ischemic brain to angiogenesis	Zhang et al. (2019)
HEK293T	Linked to RAGE-binding-piptide	Nose-to-brain delivery of AMO181a-chol and exerted neuroprotective effects	Kim et al. (2021)
	Loaded with cholesterol-modified AMO181a		
Brain endothelial cells	Rhodamine 123-loaded exosomes	the ability of exosomes to deliver drugs across the BBB	Yang et al. (2015)
MSC	MSC-exo combined with AuNPs as labeling agents	developed a method for longitudinal and quantitative *in vivo* neuroimaging of exosomes	Perets et al. (2019)
MSC	Glucose-coated gold nanoparticle (GNP) labeling and computed tomography imaging	The technique can serve as a powerful diagnostic tool for various brain disorders	Betzer et al. (2017)
HEK293	Using a slight modification of the adenovirus-free transient transfection methods	The use of exo-AAVs as an efficient gene delivery tool	Orefice et al. (2019)
Raw264.7	Loaded superparamagnetic iron oxide nanoparticles (SPIONs) and curcumin (Cur) into exosomes	Carry nanomaterials and chemical agents for simultaneous diagnosis and treatment of glioma	Zhang et al. (2019)

### 6.1 Delivery nanotechnologies

Exosomal delivery nanotechnologies are attractive because of their ability to improve the solubility and targeting specificity of natural compounds. Guo et al. enhanced the stability and solubility of quercetin by preparing quercetin-loaded exosomes. A monoclonal antibody GAP43 (a neuron-specific protein) continued to be modified on the surface of drug-loaded exosomes to alleviate neuronal damage by targeting ischemic penumbra (Guo et al., 2021). Briefly, curcumin (cur), an anti-inflammatory and neuroprotective molecule, could load macrophage (Ex-cur) and embryonic stem cell-derived exosomes (MESC-exo^cur^). They could downregulate ROS accumulation, alleviate BBB damage in lesions, reduce the expression of inflammation and the excitatory amino acid receptor, and improve neurovascular restoration (Kalani et al., 2016; He et al., 2020). Macrophage-derived exosomes containing Edaravone (Exo + Edv) could improve bioavailability, prolong t_1/2_, and have neuroprotective effects on permanent MCAO rats (Li et al., 2020b). The investigation showed that tar-exo-enkephalin (exosomes, combined with transferrin and enkephalin, were packaged into the vesicle) was capable of crossing the BBB and inhibited neuron apoptosis caused by glutamate by decreasing p53 and caspase-3 levels. This result was verified in transient MCAO rats (Liu et al., 2019b).

In addition to molecular drug delivery, nucleic acid and peptide delivery can be achieved. Pigment epithelium-derived factor (PEDF) was overexpressed in ADSCs, and PEDF-modified ADSC-derived exosomes were obtained. These ameliorated neuron OGD-induced apoptosis and I/R injury by activating autophagy (Huang et al., 2018). Yang et al. found that rabies virus glycoprotein (RVG)-modified exosomes could promote cortical neurogenesis to attenuate ischemic injury by delivering miR-124 to the infarct (Yang et al., 2017). Similarly, Yang et al. first found that nerve growth factor-Exo^RVG^ reduced ischemic injury by reducing inflammation and cell death (Yang et al., 2020). MSC-exos were conjugated to c (RGDyK) peptides and loaded with cholesterol-modified miR-210 to target the ischemic brain. Considerable improvement in angiogenesis and survival was observed in MCAO/R mice using near-infrared fluorescence imaging (Zhang et al., 2019). The receptor for advanced glycation end-products (RAGE)-binding-peptide linked to exosomes (RBP-Exo) was used for nose-to-brain delivery of anti-miRNA oligonucleotide. Moreover, compared with unmodified exosomes, RBP-Exo could downregulate RAGE more efficiently to deliver AMO181a, and reduced damage to the ischemic brain (Kim et al., 2021).

### 6.2 *In vivo* imaging and tracking

Translating exosome therapies to clinical settings is challenging and assessing treatment outcomes can only be achieved by evaluating symptom improvement, which typically takes weeks to months after treatment. Imaging of engineered exosomes allows real-time assessment of the exosomes’ fate and reveals information regarding the function, viability, and circulation of the exosomes *in vivo*. In a study, rhodamine 123-loaded exosomes were injected into zebrafish embryos, and the fluorescence of rhodamine 123 was examined in the brain tissue. The results confirmed the ability of exosomes to deliver drugs across the BBB, highlighting their potential for the treatment of brain diseases (Yang et al., 2015). Gold nanoparticles (AuNPs) are widely used in various bioanalytical and biomedical detection techniques. Perets et al. developed a method for longitudinal and quantitative *in vivo* neuroimaging of exosomes based on the superior visualization abilities of classical X-ray computed tomography (CT), combined with AuNPs as labeling agents. This technique has been proven to track the migration and homing patterns of intranasally-administered exosomes derived from MSC-exos in different brain pathologies (Perets et al., 2019). Furthermore, a previous study has established a method for non-invasive *in vivo* neuroimaging and tracking of exosomes based on glucose-coated AuNP (GNP) labeling and CT imaging. Using a mouse model of focal brain ischemia, the authors could track intranasally-administered GNP-labeled exosomes in a non-invasive manner (Betzer et al., 2017). This strategy could also be used to assess and compare the spread of exosome-enveloped adeno-associated virus (exo-AAVs) or unassociated AAVs (std-AAVs) in the brain through *in vivo* optical imaging techniques, such as probe-based confocal laser endomicroscopy (pCLE) and *ex vivo* fluorescence microscopy. The results suggest that the strategy enables tracking of exo-AAV spread and that exo-AAVs allow for widespread, long-term gene expression in the CNS, supporting the use of exo-AAVs as an efficient gene delivery tool. Moreover, a new type of engineered exosomes has been designed (Orefice et al., 2019). In this strategy, superparamagnetic iron oxide nanoparticles and Cur were loaded into exosomes and the exosomal membrane was conjugated with neuropilin-1-targeted peptides (RGERPPR peptide) using click chemistry to obtain exosomes that possess imaging and therapeutic functions, thus providing a potential approach for improving the diagnosis and treatment effects (Jia et al., 2018). Engineering can be made to achieve an efficient, targeted, or controlled release. However, precise exertion of a slow and controlled release after modification remains a great challenge. This strategy should also be compatible with cell culture conditions and must not affect the exosome itself.

## 7 Perspectives and conclusion

A key limitation for the precise characterization of EVs is the technical difficulty in isolating and characterizing pure populations of specific subtypes, as the methods currently at our disposal lead to systematic co-isolation of EVs of distinct subcellular origins. Thus, although many research articles use the term “exosome” to refer to EV preparations that have been separated from larger EVs *via* physical and biological processes (western blotting, NTA, TEM, *etc.*), it is likely that they are instead referring to a mixture of small EVs possessing both an exosomal and a non-exosomal properties. Hence, unless their MVB origin has been clearly established, using the more generic term “small EVs” is preferable.

Most research on ischemic stroke treatment and pre-development of new drugs have focused on neuron and EC development or have studied each part separately, ignoring the holistic nature of brain organization and the interactions between the parts. The NVU includes ECs, pericytes, neurons, glial cells, and ECM; this highlights the connection between the vasculature, the nerves, and the surrounding environment (Cai et al., 2017). As a prominent intercellular communication messenger, exosomes transmit biological information within the NVU and allow communication between the brain and distant tissues through the circulation of body fluids (He et al., 2021). This may explain why most neuroprotective agents were effective in preclinical studies but failed in clinical trials (Paul and Candelario-Jalil 2021). Additionally, tissue interactions in a variety of diseases, such as other neurodegenerative diseases (Guo et al., 2020), cancer (Chen et al., 2021), atherosclerosis (Zhu et al., 2019), and diabetes (Sun et al., 2018b), can be elucidated by exosomes.

miRNAs in exosomes are thought to have a substantial therapeutic impact. miRNAs are key regulatory substances carried by exosomes and have specific characteristics compared to free miRNAs. Their characteristics determine homeostasis during the treatment of ischemic stroke (Rappa et al., 2019). Moreover, the risk of microvascular occlusion is reduced in exosome therapy compared with cell therapy (Nikfarjam et al., 2020; Moghadasi et al., 2021). It has been shown that cell therapy and the use of exosomes are almost consistent. Exosomes are more stable as they show resistance to degradation and can act as a nanocarrier to deliver miRNAs and siRNAs to the CNS (Kim et al., 2019; Chen et al., 2020).

This review summarizes an abundant amount of research reporting that miRNAs in exosomes are involved in a wide range of paracrine and endocrine biological activities and fulfill important functions in different types of target cells in ischemic stroke. However, this point of view remains controversial. In general, studies examining this particular function have typically been conducted with a large excess of exosomes; these studies could not ascertain the feasibility of utilizing endogenous exosomes as functional miRNA transfer vehicles in native physiological settings (Chevillet et al., 2014). One study revealed that the number of miRNA molecules carried by the EVs is too small to make a biologically significant difference in recipient cells (Toh et al., 2018). This could be explained by one of two possibilities: either the miRNA levels in the EVs are insufficient to regulate their target mRNAs in recipient cells upon EV-mediated delivery, or the RNA-containing EVs themselves are not functional in recipient cells. Thus, it is critical to determine the effects of both the miRNA and protein on biochemical potency to reach the therapeutic dose required to elicit a relevant biochemical effect.

The protein range is rather limited when initially analyzing the protein composition of exosomes. Exosome preparations do not contain any proteins originating from the nucleus, mitochondria, endoplasmic reticulum, or Golgi apparatus. Instead, almost all identified exosomal proteins are found in the cytosol, plasma membrane, and membranes of endocytic compartments. The exosomes are formed of plasma membrane fragments as they lack abundant cell surface proteins (Théry et al., 2001; Théry et al., 2002). With the development of exosome proteomics and databases such as ExoCarta, EVpedia, and Vesiclepedia, a wealth of exosome proteins could be elucidated including different species, tissue, and uncertain cellular sources. Moreover, a previous study suggested that proteomes of MSC-derived exosomes are involved in many key biological processes that are important in cellular communication and structure; inflammation; exosome biogenesis and development; tissue repair and regeneration; and metabolism (Lai et al., 2012). Based on these studies, it can be deduced that exosome proteins have the potential to modulate many biological processes involved in disease pathogenesis and tissue repair and regeneration.

Both natural and engineered exosomes have certain targeting capabilities with the advantages of low immunogenicity (Yu et al., 2014), high membrane structure stability, low drug dose, sensitivity, and reduced drug toxicity (Yu et al., 2019; Liang et al., 2021) compared with non-biological carriers. Drug-mediated or -induced exosomes in ischemic stroke therapy are also noteworthy (Zang et al., 2020; Zhai et al., 2021); however, their targeting ability still requires improvement. Ensuring that exosomes are not disturbed by exogenous substances is also a challenge.

In most studies, only one miRNA with considerable differences in exosomes was explored. There are differences in exosome-derived miRNAs, with no uniform criteria for diagnosis and treatment of diseases. Therefore, the selected biomarker should be specific and well-established, and the exosome preparation technique should be easily repeatable. Numerous studies have analyzed miRNA sequencing in various derived exosomes after ischemic stroke or OGD/R pretreatment and found that miRNAs are differentially expressed under disease conditions (Zhang et al., 2020b; Yang et al., 2021a). Nevertheless, whether each miRNA or several mRNAs synergistically play a more effective role is yet to be elucidated. Furthermore, except for the process of diagnosis and treatment, there are several limitations that need to be overcome prior to clinical therapeutic application, and this includes a better understanding of the mechanism by which exosome therapy may lead to enhanced recovery.

Exosome manufacturing is scalable and more amenable to process optimization as the producer cells can be clonally selected and derived. Clinical focus on exosomes as natural carriers could enable nucleic acid delivery, targeted drug delivery, and non-invasive diagnosis. Therefore, it is critical to ensure standardized and reproducible production of exosomes for clinical translation. Addressing process development and scaling up exosome production would be easier if a regulatory-accepted definition of what an exosome is could be settled on. Regarding the matter, the International Society for Extracellular Vesicles provided information on upstream cell culture and downstream processing that will potentially advance exosomes toward routine manufacture. For cell culture fluid-derived exosomes, the number of cell passages, inoculation density, culture volume, and whether stimulation or other treatments have occurred, should be recorded, and details regarding medium composition and preparation, including components, such as glucose, antibiotics, growth factors, and other supplements affecting the production and composition of exosomes, should be provided in the methods. In particular, components containing exosomes, such as the serum, and cell culture history (conversion of media and adaptation steps) should also be included. For exosomes obtained from sources such as plasma, serum, and other derivatives of biological fluids, in addition to the need to control for initial volume, detailed information is required, including donor age, physiological sex, time of collection (circadian rhythm variation), diet, body mass index, specific infectious and non-infectious diseases, medications, and other factors that may affect exosome secretion (Thery et al., 2018). There are also significant downstream processing challenges to manufacturing exosomes. Different methods of preparation and purification might affect the reproducibility of obtaining exosomes (Colao et al., 2018). Although the ultracentrifugation method is the gold standard for exosome extraction, exosome purity still requires improvement. While tools such as immunomagnetic and transmission surface plasmon resonance may not fit the requirements of a large-scale purification platform, they are potentially label-free methods that may aid isolation of exosomes that can be subsequently characterized (Lobb et al., 2015; Colao et al., 2018; Whitford and Guterstam 2019).

In this review, we have included studies that investigated the application of exosomes and EVs clinically in stroke cases; however, to our knowledge, only a few studies were found (search date: 1 November 2022; Table 3). This may be due to problems with clinical sample collection and difficulty in controlling the time of onset of ischemic stroke.

**TABLE 3:** The application of exosomes and EVs in stroke clinical trials.

ClinicalTrials.gov identifier	Official tittle	Study type	First posted	Estimated/Actual study start date	Condition or disease	Intervention/Treatement
NCT05326724	The Role of Acupuncture-induced Exosome in Treating Post-stroke Dementia	Interventional (clinical trial)	13 April 2022	1 August 2022	Exosome	Device: Acupuncture
					Post-stroke Dementia	
					Acupuncture	
NCT03384433	Allogenic Mesenchymal Stem Cell Derived Exosome in Patients with Acute Ischemic Stroke	Interventional (clinical trial)	27 December 2017	17 April 2019	Cerebrovascular disorders	Biological: exosome
NCT05370105	Extracellular Vesicles as Stroke Biomarkers (EXO4STROKE)	Observational	11 May 2022	25 June 2018	Stroke Rehabilitation	Other: blood withdrawal
NCT05524506	PROgnostic Value of MicroParticles and Markers of Hemostasis in TIA and Ischemic Stroke (PROMPTS)	Observational	1 September 2022	June 2007	Brain Ischemia	-
					Cerebral Ischemia	
					Extracellular Vesicles	
					Hemostasis	
					Prognosis	

## Data Availability

The original contributions presented in the study are included in the article/Supplementary Material, further inquiries can be directed to the corresponding authors.
